# Shifts in symbiotic associations in plants capable of forming multiple root symbioses across a long‐term soil chronosequence

**DOI:** 10.1002/ece3.2000

**Published:** 2016-03-08

**Authors:** Felipe E. Albornoz, Hans Lambers, Benjamin L. Turner, François P. Teste, Etienne Laliberté

**Affiliations:** ^1^ School of Plant BiologyThe University of Western Australia35 Stirling HighwayCrawley (Perth)WA6009Australia; ^2^ Smithsonian Tropical Research InstituteApartado 0843‐03092, BalboaAnconRepublic of Panama; ^3^ Grupo de Estudios AmbientalesIMASL‐CONICET & Universidad Nacional de San LuisAv. Ejercito de los Andes 950 (5700)San LuisArgentina; ^4^ Département de Sciences biologiquesInstitut de Recherche en Biologie VégétaleUniversité de Montréal4101 Sherbrooke EstMontréalQCH1X 2B2Canada

**Keywords:** Arbuscular mycorrhizal fungi, chronosequence, ectomycorrhizal fungi, nitrogen fixation, pedogenesis, phosphorus, rhizobia

## Abstract

Changes in soil nutrient availability during long‐term ecosystem development influence the relative abundances of plant species with different nutrient‐acquisition strategies. These changes in strategies are observed at the community level, but whether they also occur within individual species remains unknown. Plant species forming multiple root symbioses with arbuscular mycorrhizal (AM) fungi, ectomycorrhizal (ECM) fungi, and nitrogen‐(N) fixing microorganisms provide valuable model systems to examine edaphic controls on symbioses related to nutrient acquisition, while simultaneously controlling for plant host identity. We grew two co‐occurring species, *Acacia rostellifera* (N_2_‐fixing and dual AM and ECM symbioses) and *Melaleuca systena* (AM and ECM dual symbioses), in three soils of contrasting ages (*c*. 0.1, 1, and 120 ka) collected along a long‐term dune chronosequence in southwestern Australia. The soils differ in the type and strength of nutrient limitation, with primary productivity being limited by N (0.1 ka), co‐limited by N and phosphorus (P) (1 ka), and by P (120 ka). We hypothesized that (i) within‐species root colonization shifts from AM to ECM with increasing soil age, and that (ii) nodulation declines with increasing soil age, reflecting the shift from N to P limitation along the chronosequence. In both species, we observed a shift from AM to ECM root colonization with increasing soil age. In addition, nodulation in *A. rostellifera* declined with increasing soil age, consistent with a shift from N to P limitation. Shifts from AM to ECM root colonization reflect strengthening P limitation and an increasing proportion of total soil P in organic forms in older soils. This might occur because ECM fungi can access organic P via extracellular phosphatases, while AM fungi do not use organic P. Our results show that plants can shift their resource allocation to different root symbionts depending on nutrient availability during ecosystem development.

## Introduction

**Figure 1:**
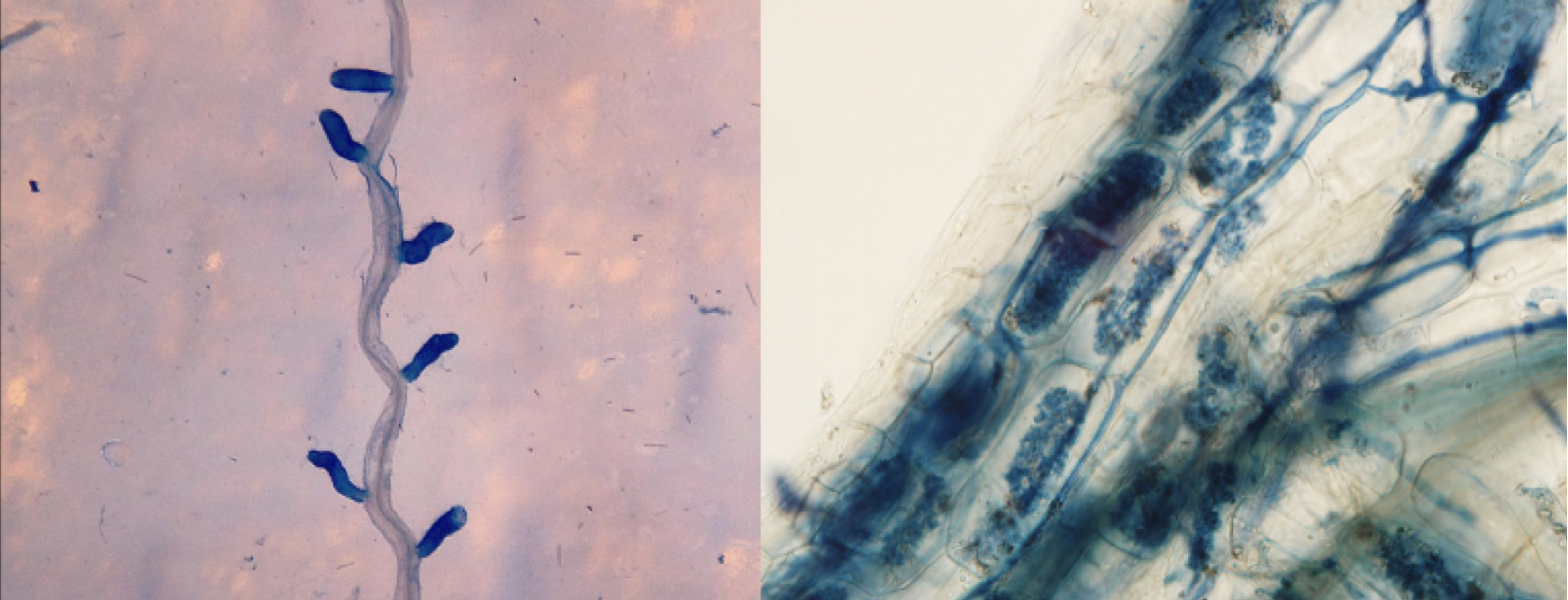
Cleared and stained roots showing arbuscular mycorrhizas (right panel) and ectomycorrhizas (left panel).

Many terrestrial plants form symbiotic associations with soil biota to enhance nutrient acquisition. The most widespread of these associations involves mycorrhizal fungi (Fig. 1), which occur in roots of >80% of all plant species (Wang and Qiu 2006; Brundrett 2009). The two main types of mycorrhizas are arbuscular mycorrhizas (AM) and ectomycorrhizas (ECM). Arbuscular mycorrhizas enhance the acquisition of inorganic phosphorus (P) and other relatively immobile nutrients, while ectomycorrhizas also allow plants to access both organic nitrogen (N) and P, as well as sorbed P (Hodge et al. 2001; Leigh et al. 2009; Plassard and Dell 2010). Some plant species also form root symbiotic associations with N_2_‐fixing bacteria in nodules, allowing plants to acquire atmospheric N (Gutschick 1984).

Plants allocate substantial amounts of carbon (C) to sustain symbiotic associations with mycorrhizal fungi or N_2_‐fixing bacteria (Pate and Herridge 1978; Smith and Read 2008). Carbon allocation to AM and ECM fungi can represent >20% of the total C fixed daily in photosynthesis (Bryla and Eissenstat 2005; Hobbie 2006). Likewise, C allocation to nodules by N_2_‐fixing plant species can represent >30% of daily photosynthates (Minchin and Pate 1973). However, plant investment in symbiotic associations depends strongly on plant nutrient requirements and soil nutrient availability (van der Heijden 2001; Lambers et al. 2008). The occurrence of AM fungi tends to be more common in neutral soils with low P availability and low organic matter content (Johnson et al. 1991; Coughlan et al. 2000; Smith et al. 2015). By contrast, ECM fungi are more common in acidic soils with lower mineral N concentrations and higher organic matter content (van der Heijden and Kuyper 2001; Lilleskov et al. 2002). Nitrogen fixation plays a greater role in N acquisition at low soil N availability and is inhibited by N fertilization (Imsande 1986; Kanayama et al. 1990). These studies suggest that plants decrease investment in root symbionts when nutrient supply is high, thus allocating C in a manner that increases acquisition of the nutrients that most strongly limit their growth.

Most plant species form associations with only one type of mycorrhizal fungi (e.g., AM or ECM). However, some plant species form dual associations with both AM and ECM fungi (Cázares and Smith 1996; Chen et al. 2000; Adams et al. 2006; Pagano and Scotti 2008), and, in some cases, a tripartite root symbiosis involves N_2_‐fixing microorganisms (e.g., *Acacia holosericea*; Founoune et al. 2002). Several studies have shown negative correlations between AM and ECM fungi, and this relationship may reflect competitive exclusion of AM fungi by ECM fungi (Lapeyrie and Chilvers 1985; Lodge and Wentworth 1990; Neville et al. 2002). On the other hand, positive relationships between nodulation and both AM and ECM colonization have been reported (Founoune et al. 2002; André et al. 2003; Lesueur and Duponnois 2005). The reliance of plants on root symbionts can be better understood by studying within‐species shifts in root symbionts with changing soil properties and plant N:P stoichiometry (Jones et al. 1998; Founoune et al. 2002; Neville et al. 2002). Such shifts have rarely been studied (but see Neville et al. 2002; Nilsson et al. 2005); hence, further research needs to identify factors involved in the balance between multiple symbioses.

Long‐term soil chronosequences (i.e., gradients of soil age) offer valuable “natural experiments” to study how soil nutrient availability and stoichiometry influence plant–soil interactions (Walker et al. 2010; Turner and Condron 2013). During tens to hundreds of thousands of years of soil and ecosystem development, changes in soil and plant communities co‐occur that strongly alter soil nutrient dynamics (Walker and Syers 1976; Wardle et al. 2004; Peltzer et al. 2010). In young soils, pH is higher, P is most abundant, and N is generally the key limiting nutrient (Walker and Syers 1976; Turner and Laliberté 2015). As soils develop, pH decreases, soil N accumulates through N_2_‐fixation, whilst P availability declines, such that N and P co‐limit plant productivity on intermediate‐aged soils (Vitousek and Farrington 1997; Laliberté et al. 2012). Additionally, while total soil P decreases during pedogenesis, its organic fraction increases and becomes the largest fraction in old soils. In strongly weathered and acidic soils, P can be strongly limiting (Vitousek and Farrington 1997; Laliberté et al. 2012) and P depletion can be sufficiently severe to cause ecosystem retrogression (Wardle et al. 2004; Peltzer et al. 2010). Soil chronosequences thus provide a unique opportunity to study changes in plant allocation to different root symbioses with decreasing nutrient availability (Treseder and Vitousek 2001).

It has been proposed that there is a community‐level shift in the relative importance of different nutrient‐acquisition strategies (specifically, the type of mycorrhizal association) during ecosystem development (Read 1991; Lambers et al. 2008). In young soils, ruderal nonmycorrhizal strategies and AM associations should be more common (Lambers et al. 2015), due to their ability to take up mineral P (Lambers et al. 2012; Smith et al. 2015). As soils age, a decrease in AM fungi in favor of ECM fungi and ericoid mycorrhizal associations should occur, because the latter can access sorbed and organic forms of P. Finally, in old severely P‐impoverished soils, nonmycorrhizal strategies should become more abundant (Lambers et al. 2008; Zemunik et al. 2015), given their highly effective strategy to acquire sorbed and organic P (Lambers et al. 2012). The validity of this model has been questioned on the basis that vegetation patterns do not follow this model in all chronosequences (Dickie et al. 2013). These models have been evaluated by observing changes in plant species composition across soil age (e.g., Zemunik et al. 2015), rather than evaluating within‐species shifts in symbiotic associations. The use of plant species capable of forming multiple symbiotic associations allows for a stronger test of these models by controlling for differences in plant host identity.

We studied changes in root symbiotic associations (AM, ECM, N_2_‐fixing nodules) within two plant species that co‐occur across contrasting stages of the Jurien Bay dune chronosequence in southwestern Australia (Laliberté et al. 2012, 2014; Hayes et al. 2014). This long‐term dune chronosequence shows a marked decrease in soil P and pH (Laliberté et al. 2012; Turner and Laliberté 2015), a shift from N to P limitation with increasing soil age (Laliberté et al. 2012; Hayes et al. 2014), and a high functional diversity in nutrient‐acquisition strategies (Hayes et al. 2014; Zemunik et al. 2015). We grew seedlings of the two focal species in soils of different ages (*c*. 0.1, 1 and 120 ka) in a glasshouse. We hypothesized that within‐species root colonization shifts from AM to ECM with increasing soil age (Lambers et al. 2008) and that nodulation in *A*. *rostellifera* declines with soil age, reflecting the shift from N to P limitation of plant growth along this chronosequence (Laliberté et al. 2012; Hayes et al. 2014).

## Materials and Methods

### Study area

The Jurien Bay dune chronosequence in southwestern Australia (30.29° S, 115.04° E) spans two million years of pedogenesis (Laliberté et al. 2012, 2014; Turner and Laliberté 2015). We focused on three stages of the chronosequence that are most contrasting in terms of the strength and type of nutrient limitation (i.e., N vs. P limitation; Table 1). The youngest dunes (~100 years) are highly calcareous and show little to no soil development (Turner and Laliberté 2015). Soils on these youngest dunes have a relatively high P availability (primarily as mineral P), but low N availability (Turner and Laliberté 2015), and plant growth on these youngest dunes is limited by N (Laliberté et al. 2012; Hayes et al. 2014). Intermediate‐aged dunes (*c*. 1000 years) are relatively high in both N and P, and plant productivity is highest and co‐limited by N, P, and possibly other nutrients (Laliberté et al. 2012; Hayes et al. 2014). Old dunes (~120 000 years) are N‐ and P‐depleted, and plant productivity is low and strongly limited by P (Laliberté et al. 2012; Hayes et al. 2014). The three dune systems correspond to chronosequence stages 1, 2, and 4 in Hayes et al. (2014) and Laliberté et al. (2014) and form a strong natural nutrient‐availability and stoichiometry gradient driven by long‐term pedogenesis (Turner and Laliberté 2015). These three chronosequence stages are <10 km apart and are exposed to the same present‐day Mediterranean climate, with a mean annual rainfall of 570 mm (Australian Bureau of Meteorology, http://www.bom.gov.au/climate/data/). They are derived from the same parent material (calcareous sand of marine origin; McArthur and Bettenay 1974; Turner and Laliberté 2015) and share the same regional species pool, with no barrier to dispersal among the different dune systems (Laliberté et al. 2014).

**Table 1:** Main soil properties for the three soil ages used. Estimated soil age, total nitrogen (N), phosphorus (P), organic phosphorus (P_org_), pH, and effective cation exchange capacity (ECEC) are from Turner and Laliberté (2015). Values are given as means ± standard error (*n *=* *10)

	Estimated soil age (ka and geological epoch)		
	0.1 (Holocene)	1 (Holocene)	120 (Middle Pleistocene)
Chronosequence stage^a^	1	2	4
Total N (g kg^−1^)	0.51 ± 0.01	1.16 ± 0.01	0.28 ± 0.01
Total P (mg kg^−1^)	351 ± 2.4	432 ± 4.8	20.3 ± 0.5
P_org_ (% of total P)	0.6 ± 0.2	3.7 ± 0.4	35.6 ± 2.4
pH (CaCl_2_)	8.2 ± 0.01	7.8 ± 0.01	5.8 ± 0.03
ECEC (cmol_c_ kg^−1^)	24.9 ± 1.5	12.9 ± 0.5	3.8 ± 0.1

a From Hayes et al. (2014)

### Species selection

We selected two native plant species that co‐occur in the three selected chronosequence stages and form at least two different types of root symbioses: (i) *Acacia rostellifera* (Benth.) Pedley (Fabaceae), which forms associations with N_2_‐fixing rhizobia as well as AM and ECM fungi (based on field sample observations), and (ii) *Melaleuca systena* Craven (Myrtaceae), which forms AM and ECM associations (Brundrett 2009), but does not fix N_2_. These species are among the few along the chronosequence that occur at these three distinct stages (Hayes et al. 2014; Laliberté et al. 2014; Zemunik et al. 2015).

### Sites selection

Turner and Laliberté (2015) used 10 sites for each chronosequence stage. In this study, we selected a representative site in each stage (i.e., close to the overall stage mean nutrient concentrations) in which both species co‐occur, to ensure compatible soil microbiota (i.e., mycorrhizal and rhizobia inoculum). These sites were Q.Y.17, Q.M.18, and S.W.35 for young, intermediate‐aged, and old stages, respectively. For each site, we collected soil from five nearby dunes located at least 200 m apart. Soil property data from all sites were obtained from Turner and Laliberté (2015) (Table 1).

### Glasshouse experiment

#### Soil collection and potting

Soils were sampled in March 2013 at each of the 15 sites, from the top 45‐cm layer. Soils were sieved (2 mm), homogenized and then dried for 5 days at 35 °C. This temperature is within the natural range for the region and was selected to ensure that soil biota would persist in the soils (Lucas et al. 1992). Soil from each site was then added to 2.8‐l pots.

Six‐month‐old seedlings were germinated and grown under specified conditions in a mixture of sterile perlite and sand without fertilizer by Men of the Trees, Hazelmere, Australia. Seedlings were transplanted into pots and watered three times a week for the duration of the experiment (6 months). At this stage, a subsample of seedlings of both species was harvested to measure initial biomass and root colonization.

#### Postharvest analyses

After 6 months of growth in the glasshouse, seedlings were harvested. Roots were severed and washed over a 1‐mm sieve immediately after harvesting to remove soil particles. Shoots and roots were oven‐dried for 3 days at 70 °C and weighed separately. A subsample of live fine roots (<2 mm diameter) was weighed, cut into 1‐ to 2‐cm segments, and stored in 10‐ml tubes for 1 week at 5 °C. Dry weight of the subsample was later estimated by calculating root water content. Roots were cleared using potassium hydroxide (10% w/v) for five hours in a water bath at 90 °C. Following clearing, we used an ink–vinegar solution to stain roots (Vierheilig et al. 1998). Finally, cleared and stained roots were placed in a 50% (v/v) lactoglycerol solution for storage.

Root colonization was determined following the gridline intersect method (Giovannetti and Mosse 1980) at 200× magnification, counting intersects that had arbuscules or vesicles for AM fungi, a mantle for ECM fungi, or a Hartig net when the mantle was absent. The presence of hyphae was not counted when the other structures were absent to ensure that other endophytes were not counted as AM fungi. We counted at least 130 intersections for each sample. For *A. rostellifera*, all nodules were collected, oven‐dried at 70°C for 48 h and weighed.

Leaf samples were digested in a mixture of sulfuric and salicylic acid and hydrogen peroxide (i.e., Kjeldahl digest), with N and P detection by automated colourimetry using a Technicon AutoAnalyzer II (Technicon Instruments Corp., Tarrytown, NY). Initial dry biomass (B) of planted seedlings was estimated through an allometric regression equation based on seedling height (H) and stem diameter (D) using additional seedlings for each species (*A. rostellifera*: ln(B) = 0.75 ×  ln(D^2^ × H) − 2.69, *R* ^2^ = 0.81, *n *=* *20; *M. systena*: ln(B) = 0.52 ×  ln(D^2^ × H) – 2.31, *R* ^2^ = 0.80, *n *=* *20). The relative growth rate (RGR) was calculated (Hunt 1982). We also recorded initial mycorrhizal colonization of rehydrated oven‐dried roots, using methods described above; initial mycorrhizal root colonization was either absent or low: for *A. rostellifera,* it was 0.4% and 0% for ECM and AM, respectively; for *M. systena,* it was 1% and 0.4%, respectively. We tested the effects of root rehydration on mycorrhizal colonization; extraradical hyphae were lost, but percent root length colonization estimates was not affected (paired *t*‐test; *P *≥* *0.4; Table S1).

### Statistical analyses

We used linear mixed‐effect models (Pinheiro and Bates 2001) to test for differences in mycorrhizal colonization, nodule biomass, RGR, and leaf N and P concentrations among plant species and chronosequence stages, including the interaction between these two fixed factors. Additionally, we tested for a potential effect of ECM colonization on nodulation and AM colonization using a linear model, with chronosequence stage as a covariate. Site was specified as a random effect, because more than one sample came from each site. In all analyses, residuals were inspected visually to check model assumptions. When models did not meet assumptions (i.e., residuals centered around zero and homoscedasticity), appropriate variance structures were specified in a second model, and both models were compared using the Akaike Information Criterion (AIC) and likelihood ratio tests (Zuur et al. 2009). When a main term was significant, *post hoc* Tukey tests were performed (Hothorn et al. 2008). All analyses were conducted in R (R Core Team 2015) using the “nlme” (Pinheiro et al. 2010) and “multcomp” (Hothorn et al. 2008) packages.

## Results

### Mycorrhizal colonization

**Figure 2:**
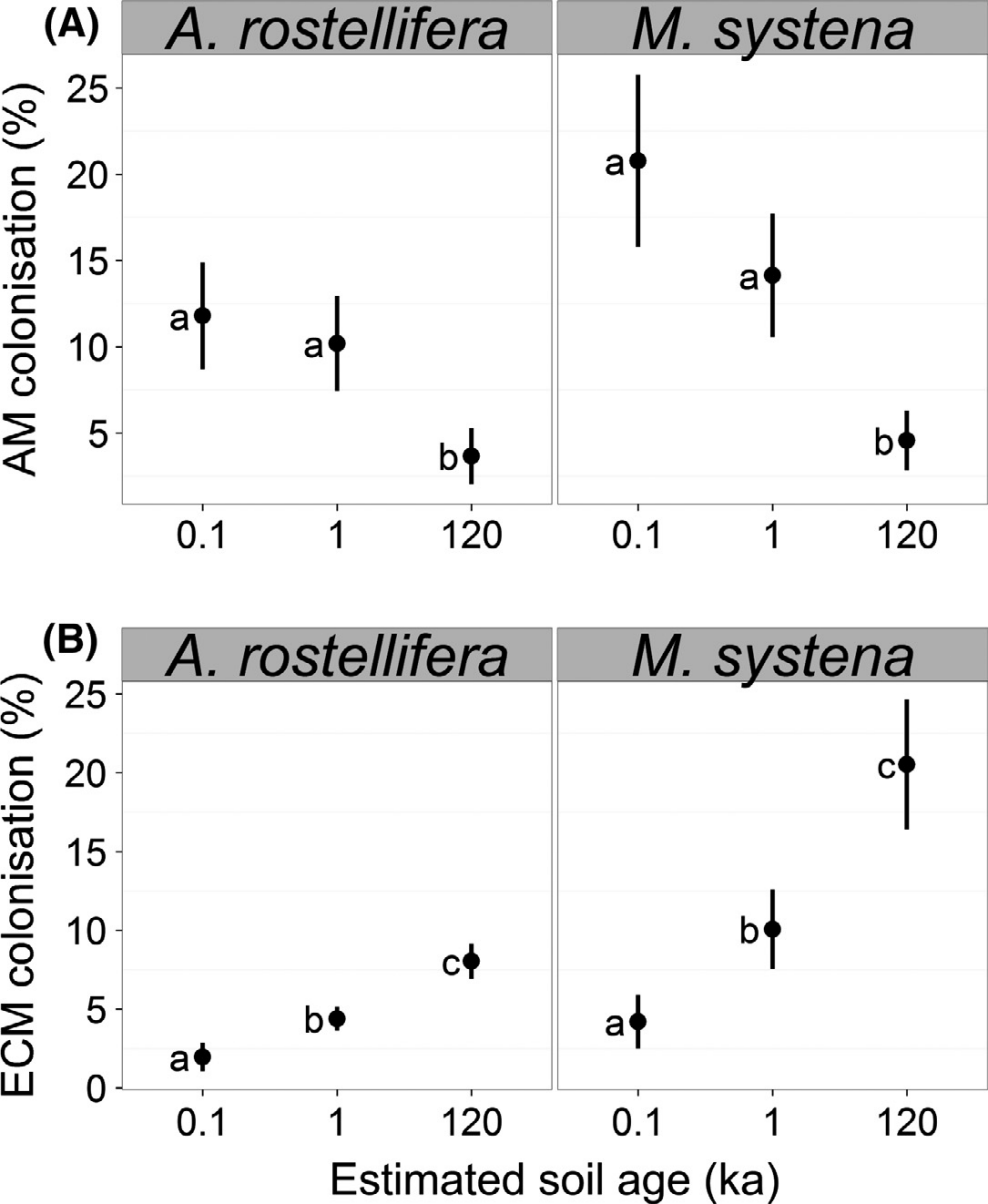
Percentage of root length colonized by (A) arbuscular mycorrhizal fungi (AM; percentage of grid intercepts) and (B) ectomycorrhizal fungi (ECM; percentage of root tips) for *Acacia rostellifera* and *Melaleuca systena* with increasing soil age. Means and 95% confidence intervals (CI) are shown. Different letters indicate significant (*P *≤* *0.05) differences among soil ages based on *post hoc* Tukey tests. Correction statement: [Correction added on 18 March 2016, after initial online publication. Figure 2 is now corrected in this version.]

Changes in root colonization by AM fungi differed between species, but these differences depended on soil age (species × stage interaction; *P *≤* *0.05; Fig. 2A; Table S2). Arbuscular mycorrhizal root colonization was greater in *M. systena* than in *A. rostellifera* only in the young soils (*P *≤* *0.02), while there were no differences in AM root colonization between the two species in either intermediate‐aged or old soils (*P *≥* *0.4). Arbuscular mycorrhizal root colonization of both species was greatest on the youngest and intermediate‐aged soils, and least on the oldest soils (*P *≤* *0.001).

Both species showed similar patterns of increasing ECM colonization with increasing soil age, although differences varied between species (species × stage interaction; *P *≤* *0.001; Fig. 2B; Table S2). *Melaleuca systena* generally showed greater ECM colonization than did *A. rostellifera* (*P *≤* *0.001), with the exception of the youngest soil, where the species showed similarly low ECM colonization (*P *≥* *0.16). Also, we found no significant relationships between AM and ECM colonization for both species when soil age was taken into account (*P *≥* *0.79).

### Nodule biomass in *Acacia rostellifera*

**Figure 3:**
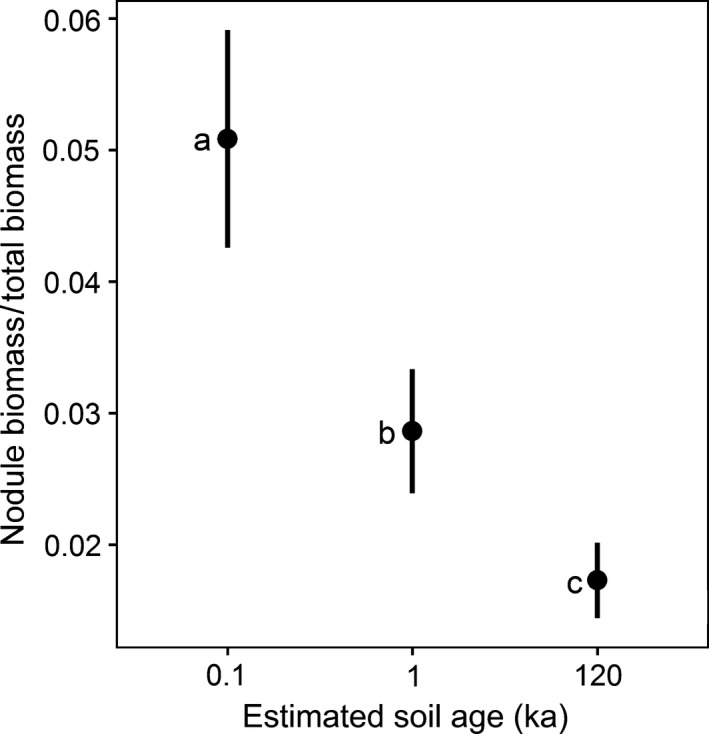
Relative investment in nodules (i.e., ratio between nodule biomass and total plant biomass) of *Acacia rostellifera* seedlings with increasing soil age. Means and 95% confidence intervals (CI) are shown. Different letters indicate significant (*P *≤* *0.05) differences among chronosequence stages based on *post hoc* Tukey tests.

Total seedling (*P *≤* *0.001) and nodule biomass in *A. rostellifera* declined with increasing soil age (*P *≤* *0.001). Therefore, we measured the relative investment in N_2_‐fixing nodules in *A. rostellifera* as the ratio between nodule biomass and total plant biomass. This ratio also declined with increasing soil age (*P *≤* *0.001; Fig. 3; Table S2). We found no correlation between relative nodule production and ECM root colonization after controlling for differences in soil age (*P *≥* *0.98). There was also no effect of AM colonization on nodule biomass (*P *≥* *0.87).

### Leaf nutrient concentrations and biomass

**Figure 4:**
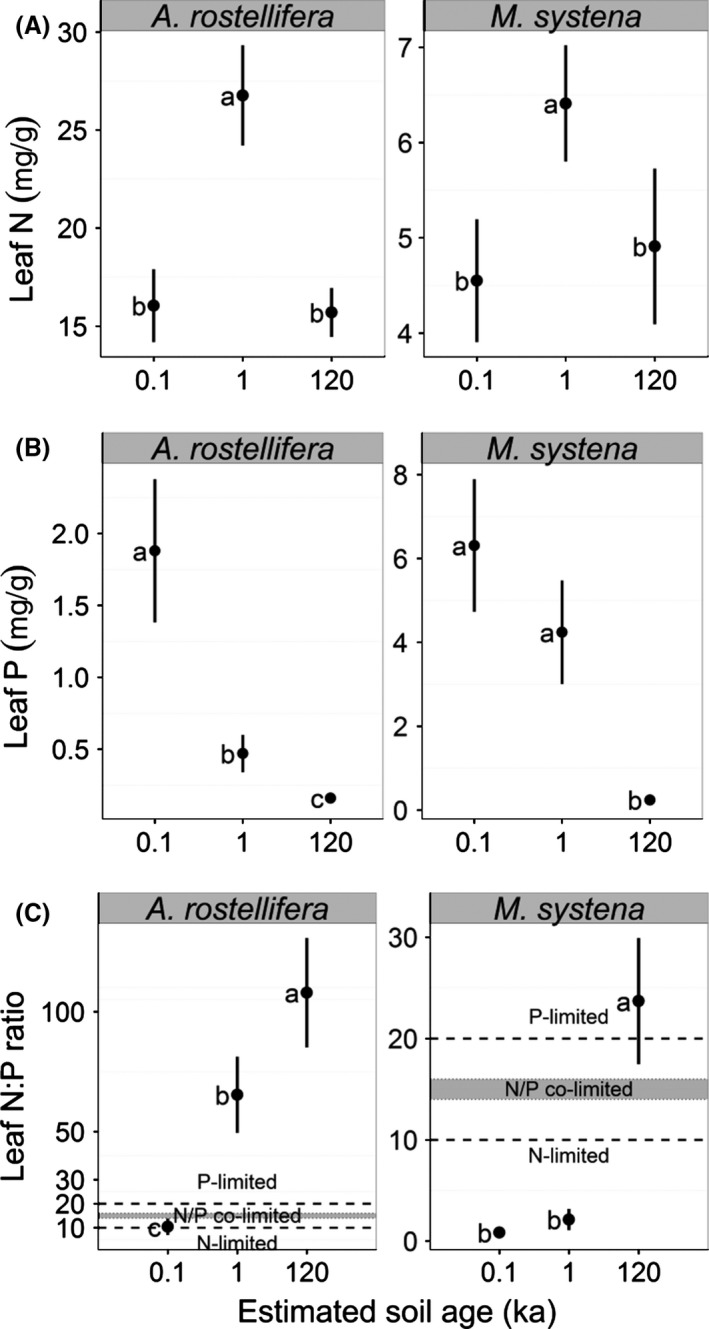
(A) Leaf nitrogen (N) and (B) phosphorus (P) concentrations and (C) N:P ratio of *Acacia rostellifera* and *Melaleuca systena* with increasing soil age. Means and 95% confidence intervals (CI) are shown *n *=* *10. Different letters indicate significant (*P *≤* *0.05) differences among soil ages based on *post hoc* Tukey tests. Black dashed lines indicate thresholds for N or P limitation, following Güsewell (2004). Gray area indicates thresholds for N or P limitation based on Koerselman and Meuleman (1996).

Leaf [N] followed a similar pattern for both species across the chronosequence, being highest on intermediate‐aged soils (*P *≤* *0.003; Fig. 4A; Table S2), where soil total [N] was highest (Table 1). Leaf [N] was higher in *A. rostellifera* than in *M. systena* across all soil ages (*P *≤* *0.001). Leaf [P] decreased from young to old soils for both species (*P *≤* *0.001; Fig. 4B; Table S2). Leaf [P] was lower in *A. rostellifera* than in *M. systena* on young and intermediate‐aged soils (*P *≤* *0.001), but on old soils both species had similarly low leaf [P] (*P *≥* *0.2). Leaf N:P ratio increased from young to old soils in both species (*P *≤* *0.001; Fig. 4C; Table S2). On intermediate‐aged soils, the N:P ratio of *A. rostellifera* (65 ± 7.9) pointed toward P limitation, while that of *M. systena* (2.1 ± 0.5) pointed toward N limitation.

**Figure 5:**
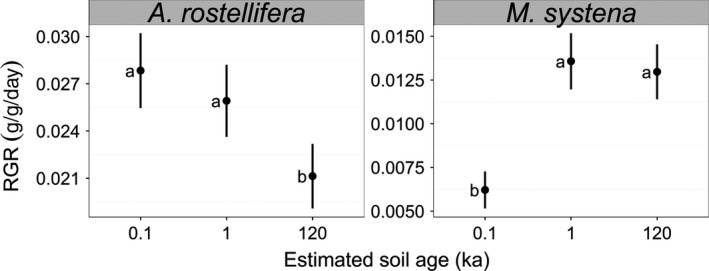
Relative growth rate (RGR) of *Acacia rostellifera* and *Melaleuca systena* seedlings grown on soils of different ages. Means and 95% confidence intervals (CI) are shown. Different letters indicate significant (*P *≤* *0.05) differences among soil ages based on *post hoc* Tukey tests.

There were differences in RGR between species, but these depended on soil age (species × stage interaction; *P *≤* *0.01; Fig. 5; Table S2). The RGR of *A. rostellifera* was greater on both the youngest and intermediate‐aged soils than on the oldest soils (*P *≤* *0.02), while for *M. systena* it was greatest on intermediate‐aged and old soils (*P *≤* *0.01), and lowest on the youngest soils (*P *≤* *0.01).

## Discussion

### Shifts in mycorrhizal colonization

Consistent with our hypothesis, root colonization by AM fungi declined with increasing soil age, whereas previous studies have found AM colonization increasing with declining soil P availability (Abbott et al. 1984; Bentivenga and Hetrick 1992; Treseder and Vitousek 2001). However, these studies were conducted at higher soil [P] and across a much smaller soil [P] range (Francis and Read 1994) than that along the studied chronosequence (Turner and Laliberté 2015). In addition, these studies used species that only form AM, whereas our study focused on species forming multiple associations simultaneously. Furthermore, soil pH decreased and previous studies have shown that AM fungi tend to dominate on young alkaline‐to‐neutral soils (Piotrowski et al. 2008; Zangaro et al. 2012), and soil pH <5 can decrease AM colonization (Clark 1997; Coughlan et al. 2000). In our study, pH declined to only 5.8 in the oldest soils, suggesting that pH inhibition likely did not contribute to the effect of soil age on AM colonization. Our results suggest that AM associations are favored in younger soils where most P is in mineral forms (Lambers et al. 2008; Turner and Laliberté 2015).

Root colonization by ECM fungi was about four times greater in the oldest soils than in the youngest soils for both species. Although the oldest soils had a much lower total [P], organic P represented a much larger fraction. Ectomycorrhizal fungi are efficient at accessing organic forms of N and P (Read 1989; Antibus et al. 1992; Chalot and Brun 1998). Consequently, ECM colonization may be related to the organic soil P fraction, consistent with results of Harvey et al. (1976). Old acidic soils might be better suited for ECM fungi than young alkaline soils (Piotrowski et al. 2008; Zangaro et al. 2012), as the optimum conditions for ECM fungi are between pH 4 and 5 (Aggangan et al. 1996; Yamanaka 2003). Young soils in our study exhibited a pH between 5.8 and 8.2, suggesting that the decline in pH contributed to the increase of ECM fungi with increasing soil age. However, we cannot disentangle potential effects of total P from those due to pH, because total P and pH decline simultaneously during pedogenesis.

Negative relationships between AM and ECM have been interpreted as competitive exclusion of AM fungi by ECM fungi (Chen et al. 2000; Adams et al. 2006). Similarly, colonization shifts from AM to ECM with soil depth have been found (Neville et al. 2002), with higher ECM colonization in upper soil layers, where organic matter content is greater. In coniferous forest, AM fungi dominate in nutrient‐rich soils with high pH, while ECM fungi dominate in soils with low nutrient availability and lower pH (Nilsson et al. 2005). The lack of a relationship between AM and ECM at any soil age in our study suggests that the observed shift from AM to ECM colonization was driven by changes in soil properties, rather than reflecting a direct negative effect of ECM fungi on AM fungi.

### Shifts in nodule biomass

Nodulation in *A. rostellifera* declined with increasing soil age, likely because plant growth on the oldest soils is limited by the availability of P, rather than N. Nodulation might be constrained in old soils by the relatively high P demand of N_2_ fixation (Sprent and Raven 1985; Sprent 1999; Raven 2012). Thus, on old soils, where both N and P availability are extremely low, legumes might acquire N predominantly via ECM, rather than rhizobia. There was no relationship between nodulation and ECM colonization in *A. rostellifera* once differences in soil age were controlled for. These results differ from those obtained by Diagne et al. (2013), who found that ECM fungi promote nodulation under P limitation in *A. mangium*. However, Diagne et al. (2013) used soils with relatively high P levels (4.8 mg Olsen P kg^−1^), while resin [P] in our study ranged between 0.6 and 3 mg kg^−1^ (Turner and Laliberté 2015). Furthermore, previous studies have shown that a soil pH <4.5 can be detrimental for the two main N_2_‐fixing rhizobia (*Rhizobium* and *Bradyrhizobium*; Graham 1992; Graham et al. 1994). As soil pH in the present study ranged from 8.2 to 5.8, the decrease in nodulation is likely related to nutrient limitation, rather than a low soil pH.

### Shifts in the type and strength of nutrient limitation

Both leaf [N] and leaf [P] reflect the low availability of these nutrients in soils (Laliberté et al. 2012; Turner and Laliberté 2015). Furthermore, leaf N:P ratio increased more than 10‐fold for *A. rostellifera* and 20‐fold for *M. systena* from the youngest to the oldest soils, consistent with shifts from N limitation to strong P limitation of plant productivity along the chronosequence (Laliberté et al. 2012; Hayes et al. 2014). Leaf N:P increased markedly in *A. rostellifera* between the youngest and intermediate‐aged soils, while there was no difference between N:P on these two soil ages for *M. systena*. The change in *A. rostellifera* was associated with a greater increase in leaf [N], presumably due to its N_2_‐fixation capacity. Foliar N:P in a N_2_‐fixing shrub is also low on young soils along a 120 000 year chronosequence in New Zealand (Richardson et al. 2004), due to high leaf [N] rather than low leaf [P].

The shifts in mycorrhizal colonization with increasing soil age could be due to changes in inoculum potential, which decreases with increasing soil age for AM fungi, but increases with soil age for ECM fungi (Piotrowski et al. 2008; Zangaro et al. 2012). However, such changes in inoculum potential might be related to longer‐term feedback between plants and soil biota that ultimately depend on soil nutrient availability. Additionally, soils in this study were sieved and dried at nondetrimental temperatures (Lucas et al. 1992), yet this could have potentially removed fungal species that colonize through hyphae. Future experiments should aim to disentangle the role of such biotic and abiotic effects on the balance of multiple symbioses, to assess the effects of soil abiotic properties and inoculum potential independently.

In conclusion, our results show within‐species shifts between different root symbiotic associations during long‐term soil and ecosystem development, consistent with those predicted by Read (1991) and Lambers et al. (2008). This might be associated with a shift from N to P limitation of primary plant productivity, soil pH or inoculum potential (Nilsson et al. 2005; Zangaro et al. 2012). Our study supports the hypothesis that the importance of different mycorrhizal types changes with soil age (Lambers et al. 2008). Our results on intraspecific shifts in nutrient‐acquisition strategies complement those of a recent study along the same chronosequence showing that, at the community level, ECM plants become more abundant as soils age (Zemunik et al. 2015). Further work on within‐species shifts in symbiotic associations and their functional significance is needed to better understand the role of mycorrhizal fungi during long‐term ecosystem development (Dickie et al. 2013).

## Conflict of Interest

The authors declare that they have no conflict of interest.

## Data Accessibility

All data are included in the manuscript and supporting information.

## Supporting information


**Table S1.** Comparison of mycorrhizal root colonization between fresh and rehydrated roots. Values shown as mean ± SE based on paired t‐test

**Table S2.** Summary of statistical outputs. Values shown are degrees of freedom (DF), F‐test and p‐value of individual mixed‐effect models of two factors (Stage and Species), and their interaction for each variable.

**Table S3.** Data file used in this study with plant biomass, N and P concentration, AM and ECM root colonization, and nodule biomass.
