# Supplementary material for: Shifts in symbiotic associations in plants capable of forming multiple root symbioses across a long‐term soil chronosequence
Source: Ecol Evol. 2016 Mar 8;6(8):2368–77. doi: 10.1002/ece3.2000 (PMC4782245; doi:10.1002/ece3.2000)
Supplement: Supplementary file 1 — Table S1. Comparison of mycorrhizal root colonization between fresh and rehydrated roots. Values shown as mean ± SE based on paired t‐test [file ECE3-6-2368-s001.docx]

**Table S1** Comparison of mycorrhizal root colonisation between fresh and rehydrated roots. Values shown as mean ± SE based on paired t-test

| Fresh roots | | Rehydrated roots | P-value |
| --- | --- | --- | --- |
| *Acacia rostellifera* | | | |
| ECM colonisation (%) | 6.5 ± 1.9 | 6.2 ± 1.1 | 0.7 |
| AM colonisation (%) | 3.1 ± 0.9 | 3.3 ± 0.6 | 0.8 |
| *Melaleuca systena* | | | |
| ECM colonisation (%) | 18.8 ± 1.0 | 20.3 ± 1.3 | 0.4 |
| AM colonisation (%) | 2.7 ± 0.7 | 2.4 ± 0.8 | 0.7 |
