# Supplementary material for: Shifts in symbiotic associations in plants capable of forming multiple root symbioses across a long‐term soil chronosequence
Source: Ecol Evol. 2016 Mar 8;6(8):2368–77. doi: 10.1002/ece3.2000 (PMC4782245; doi:10.1002/ece3.2000)
Supplement: Supplementary file 2 — Table S2. Summary of statistical outputs. Values shown are degrees of freedom (DF), F‐test and p‐value of individual mixed‐effect models of two factors (Stage and Species), and their interaction for each variable. [file ECE3-6-2368-s002.docx]

**Table S2** Summary of statistical outputs. Values shown are degrees of freedom (DF), F-test and p-value of individual mixed-effect models of two factors (Stage and Species) and their interaction for each variable.

|  | Stage | | | Species | | | Interaction | | |
| --- | --- | --- | --- | --- | --- | --- | --- | --- | --- |
|  | DF | F-test | p-value | DF | F-test | p-value | DF | F-test | p-value |
| AM colonisation (%) | 2 | 15.74 | 0.0004 | 1 | 10.51 | 0.0023 | 2 | 4.77 | 0.0136 |
| ECM colonisation (%) | 2 | 36.35 | <.0001 | 1 | 6.75 | 0.0129 | 2 | 11.21 | 0.0001 |
| Nodulation | 2 | 35.21 | <.0001 | - | - | - | - | - | - |
| RGR (g g^-1^ d^-1^) | 2 | 10.16 | 0.0026 | 1 | 278.32 | <.0001 | 2 | 28.3 | <.0001 |
| Leaf N (mg g^-1^) | 2 | 14.96 | <.0001 | 1 | 545.51 | <.0001 | 2 | 21.1 | <.0001 |
| P (mg g^-1^) | 2 | 53.38 | <.0001 | 1 | 8.72 | 0.005 | 2 | 31.54 | <.0001 |
| Leaf N:P ratio | 2 | 50.63 | <.0001 | 1 | 65.44 | <.0001 | 2 | 40.37 | <.0001 |
| Nodulation was calculated as total nodule biomass divided by total seedling biomass. | | | | | | | | | |
